# Development of Sciatic Neuropraxia following Abdominal Surgery in 3 Giant Breed Dogs

**DOI:** 10.1155/2021/5313684

**Published:** 2021-09-27

**Authors:** Laura Marti, Julia P. Sumner

**Affiliations:** Department of Clinical Sciences, College of Veterinary Medicine, Cornell University, Ithaca, New York, USA

## Abstract

This report describes the clinical course of three giant breed dogs (2 Great Danes and 1 Saint Bernard) that developed sciatic neuropraxia following successful surgical management of gastric dilatation and volvulus (GDV). All three patients received physical rehabilitation with varying degrees of success. Two patients died of unrelated causes within a year of their initial presentation. The third case recovered nerve function and is alive with minimal neurologic deficits at the time of publication. This paper is aimed at positing potential causes for this complication and highlighting the importance of proper management of giant-breed dogs during hospitalization. Special attention should be given in regards to intraoperative positioning and postoperative care including frequent walks or changes in positioning, deep kennel bedding, and physical therapy.

## 1. Introduction

Postoperative neuropraxia is a well-recognized complication in humans undergoing surgery [1–3]. A recent estimate indicates that nearly one-third of all human medicolegal cases involving a patient undergoing anesthesia were related to the acquisition of nerve injury [1]. This complication is typically associated with orthopedic surgery on the affected limb in human patients, though spinal and abdominal surgeries have also been implicated [2].

Nerve injury results from one or more pathologic forces, such as stretch or compression, acting on the nerve in such a way that normal function is disrupted [1]. The disruption in function can nearly always be attributed to ischemia or mechanical injury [3]. Peripheral nerve injury is a well-documented phenomenon in veterinary medicine, particularly among large animals. Prolonged recumbency has been implicated in compression nerve injury with “downer cows” [4, 5]. Similarly, postanesthetic neuropathy in horses is theorized to be related to factors including patient positioning and ischemia secondary to inadequate blood pressure maintenance [6]. In contrast, peripheral nerve injuries in small animals are more closely associated with direct intraoperative damage rather than perioperative conditions [7].

In this report, we describe three cases of giant breed dogs that developed sciatic neuropraxia following abdominal surgery, likely due to intraoperative positioning and prolonged recumbency in the postoperative period. This case report is aimed at exposing this potential risk of developing peripheral nerve injury for giant-breed dogs and at highlighting recommendations based on current literature that may reduce the risk of peripheral nerve injury from occurring.

## 2. Case Presentation

### 2.1. Case 1

**Figure 1:**
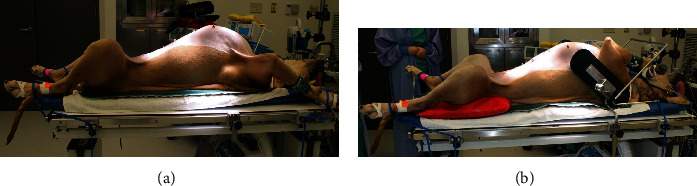
(a) An example of Great Dane in standard dorsal recumbency positioning prior to surgical procedure. (b) Note the additional supports and padding aimed at keeping the patient in the correct position without applying abnormal force to the limbs.

An eight-year-old, female spayed Great Dane was presented to the emergency service of a referral hospital for suspected gastric dilatation with volvulus (GDV). The patient was recumbent and minimally responsive upon presentation. The patient had a history of osteoarthritis in both stifles that were being managed medically, and medial buttress that was palpated bilaterally, but no previous history of neurological deficits. Radiographs confirmed the presence of GDV, and emergency surgery was performed to correct the volvulus and perform a gastropexy. The dog was placed in dorsal recumbency for the duration of surgery and secured to the operating table in a standard fashion using four limb ties (Figure 1(a)). The dog was critically ill following surgery and remained recumbent for the remainder of the night.

The following day, the patient was moribund and reluctant to rise. When assisted to stand, conscious proprioceptive deficits present in the left hind limb caused knuckling of the left hind foot when walking, regardless of the surface. A neurological examination found absent conscious proprioception, delayed to absent hopping, and absent withdrawal in the left hind limb. The neurologic lesion associated with these findings was characterized as a left sciatic neuropathy. The prioritized differential diagnosis was sciatic neuropathy secondary to compression or stretch.

The dog was referred to the sports medicine service, which recommended a rubber boot to protect the left hind foot from injury. The patient also underwent electroacupuncture treatment while hospitalized. She was discharged 10 days after surgery, with instructions to perform a range of home exercises to improve weight bearing, weight shifting, and range of motion in the affected limb. One week following surgery, the patient was noted by the owners to be walking with intermittent correct placement of the left hind limb.

Repeat neurological examination found an absent withdrawal, conscious proprioceptive deficits, and muscle atrophy in the left hind limb. Small abrasions were also noted between the digits and on the dorsum of the paw of the left hind. The abrasions were bandaged, and the dog was fitted with a brace (Therapaw Toes-up Dorsiflex Assist, Lebanon, NJ, USA).

Approximately three months following presentation, the dog returned for a recheck examination and initial underwater treadmill session. Her owners reported that the dog was ambulating well with the brace. Upon examination, the patient showed some improvement with an incomplete withdrawal on the left hind limb, but conscious proprioceptive deficits remained. The abrasions on the paw had healed. A list of recommended home exercises was provided to the owners. The owners cancelled the two- and four-week recheck appointments due to improvement of the clinical signs. The following month, four months after the initial presentation, the patient's health rapidly deteriorated and she passed away at home. No postmortem examination was performed, and the exact cause of death remains unknown.

### 2.2. Case 2

An eight-year-old female spayed Great Dane was presented to the emergency department of a referral hospital for treatment following radiographs confirming the presence of GDV and was weak and unwilling to walk upon examination. The owners reported the dog had been uncoordinated recently and had difficulty navigating stairs. The patient underwent emergency surgery to correct the volvulus, and an incisional gastropexy was performed. Positioning for surgery was in standard dorsal recumbency with the dog secured to the table with four limb ties. Due to concerns for nerve damage, it was recommended that she was walked every four hours postoperatively and the side on which she was lying was changed frequently to avoid pressure sores.

The patient was unable to be walked frequently on the night following surgery as she was unwilling to rise, and there was an insufficient labor force to lift her. The following morning, the patient was reluctant to walk and had mild knuckling of the left hind limb. Two days after presentation, the patient was noted to have sores on both elbows and a wound on the left hind foot. Her paresis progressed, and she was unable to walk without assistance. Proprioception was absent in both hind limbs. A neurologic examination found ambulatory to nonambulatory tetraparesis with severe knuckling of the left hind limb. Hopping was mildly delayed in the left forelimb and severely delayed to absent in the hind limbs. Withdrawal reflex was decreased in the left hind leg, but patellar reflexes, cutaneous trunci, and perineal reflexes were all within normal limits. Palpation revealed mild caudal lumbar pain. Deficits were localized to the left sciatic nerve and C1 to C5 segments of the spinal cord. The top differential was cervical spondylomyelopathy that was exacerbated due to positioning under anesthesia; however, the asymmetry of the lesions is an atypical presentation of the condition. Therefore, neuropraxia of the left sciatic nerve was also diagnosed, likely due to positioning during surgery and/or prolonged recumbency during recovery. The patient was started on steroid therapy (1.7 mg/kg prednisone PO q 24 h for two weeks, followed by 1.7 mg/kg PO q48h for two weeks) to assist in resolving inflammation in the spinal cord associated with the myelopathy.

At reevaluation six days after surgery, the patient had subjective improvement in her ability to walk with the help of a harness, but still required at least 70% assistance. She was referred to the sports medicine service, which described their findings of nonambulatory tetraparesis, knuckling of the left hind limb, crossing over in both the front and hind limbs, reduced withdrawal reflex, conscious proprioceptive deficits in all limbs, and evidence of dorsal scuffing. The patient was painful at the level of C6 with left lateral flexion. Ultimately, she was fitted with a custom orthotic brace. Three months following the initial presentation, the owners expressed concern that the dog was falling and was weaker. Ten days later, the patient was seen for an intestinal obstruction secondary to a foreign body. Due to her deteriorating condition, the owners opted for humane euthanasia.

### 2.3. Case 3

An eight-year-old female spayed Saint Bernard dog presented to the emergency department of a referral hospital for evaluation of nonproductive retching and lethargy. Abdominal radiographs confirmed the presence of GDV, and emergency surgery was performed to correct the GDV and perform an incisional gastropexy. No history of prior neurologic disease was noted by the owner. The patient recovered uneventfully from anesthesia. The dog was placed in a large kennel with deep bedding, and instructions were to walk or change the dog's position every four hours.

Twelve hours later, significant gait abnormalities involving the right pelvic limb and a small (approximately 1 cm) wound on the dorsum of the right paw were noted. A neurological examination indicated paresis of the right pelvic limb and conscious proprioceptive deficits leading to knuckling and dragging of the right hind foot. The right hind limb had a mild plantigrade stance and notably decreased hopping compared to the left hind limb. The withdrawal reflex was decreased on the right pelvic limb, but the lateral digits elicited a better response than the medial digits. Patellar reflexes were intact bilaterally. The patient's recovery plan was updated to include more frequent leash walks, and a crib mattress was placed in her cage to minimize the potential compression of the sciatic nerve while recumbent. A sports medicine consultation had similar findings to the neurologic examination. The patient was discharged four days after admission with instructions for rehabilitation exercises to perform at home. The patient returned two days later for a recheck of the neuropraxia and for a bandage change on the right hind foot. The paresis of the right hind limb noticed previously was improved, and no knuckling-over was seen. The right tarsus remained dropped compared with the left, but withdrawal reflexes were present in both hind limbs. The owners noted significant improvement with the patient's stability. She was discharged with instructions to return if complications arose with the wound on the dorsum of the right hind paw. Six weeks following discharge from the hospital, the owners felt that the dog's disposition and behavior had returned to normal. They reported the sciatic neuropraxia had improved significantly, and the patient's mobility was back to near normal within eight weeks of discharge. Follow-up correspondence with the owners nine months after surgery indicated the patient was doing well as of the time of writing.

## 3. Discussion

Sciatic neuropraxia is an uncommon complication following a surgery not involving the pelvis or pelvic limbs. Much of the recorded data on sciatic neuropraxia in companion animals is focused on patients that have direct accidental trauma during an orthopedic or soft tissue procedure or a regional intramuscular injection [7–10]. In humans, the development of a lower limb neuropathy following abdominal surgery is a documented but rare phenomenon. One study found only 0.17% of abdominal surgeries had a postoperative nerve complication in the lower extremity, with the majority involving the femoral nerve [2]. In this study, nerve injury was attributed to compression of the femoral nerve in the region of the pelvis, either through positioning or use of self-retaining retractors [2]. Multiple factors contribute to potential nerve injuries, including duration and magnitude of the force causing the injury as well as the size and structure of the nerve affected [2]. Prolonged recumbency has been implicated in the development of secondary nerve damage in downer cows, likely as a result of increased pressure [4, 5]. Given the size of the dogs involved, and the severity of their illness, prolonged periods of recumbency likely contributed to nerve damage in these cases.

Nerve injuries are generally classified by location and severity and exist on a continuum of severity which reflects the likelihood of returning to normal function following healing [2]. The sciatic nerve is particularly susceptible to damage at different points in its course down the limb. The peroneal branch is vulnerable around the stifle where it separates from the tibial branch and angulates where it passes between muscles on the cranial crus in a relatively superficial position [8]. In addition, the peroneal branch has fewer and larger funiculi with less connective tissue support compared to the tibial nerve [7]. The peroneal nerve's organization means that any force applied to the nerve itself will concentrate on the funiculi rather than connective tissue, compared to a nerve with smaller funiculi or more connective tissue [7]. These factors increase the risk of compression forces acting upon it and causing significant disruption to the function of the nerve [7].

Intraoperative positioning potentially contributed to neuropraxia in these cases, and there is evidence in human medical literature to support this. Human patients positioned in the lithotomy position, on their backs with the legs elevated and bent, for extended periods of time are at increased risk of developing postoperative neuropathy [1]. It is worth noting that the lithotomy position is similar to the position of a canine patient in dorsal recumbency for an abdominal procedure (Figure 1). In the awake patient, discomfort caused by such positioning would prompt adjustment of body position to relieve the sensation. However, anesthesia impairs this ability making the patient more susceptible to injury due to uncomfortable positioning. Additionally, positioning is only one aspect of patient management, and determining the correct position is an imprecise science. A case report describes a Japanese sumo wrestler who underwent spinal surgery [11]. The day prior to the procedure, the patient was positioned as he would be under anesthesia and remained there for twenty minutes. The patient reported no discomfort during that time, but subsequently developed a brachial plexus compression injury believed to be secondary to compression by the pectoral muscles during the procedure [11]. This case illustrates that patient variables must be considered. The wrestler's unique physical conformation resulted in injury despite careful consideration of patient positioning. Additionally, thin body condition has been implicated in an increased likelihood of perioperative nerve injury [1, 3].

Given these findings, it is reasonable to suggest that care should be taken when manipulating and positioning these dogs under general anesthesia. The surgical procedure may necessitate positioning in dorsal recumbency; however, certain precautions can be taken to prevent the occurrence or exacerbation of peripheral nerve injury (Figure 1(b)). Across several surveys, a major concern with regards to surgical positioning is hyperextension and hyperflexion of joints. The American Society of Anesthesiologists surveyed members, and while there is not enough information to conclusively link joint malpositioning with peripheral nerve injuries, the majority of respondents indicated that limiting hip flexion and hamstring stretching while positioning patients would likely limit the occurrence of perioperative sciatic neuropraxia [1]. Increased padding is also a common recommendation, on the basis that it may prevent pressure damage to nerves, especially the peroneal branch where it crosses the fibular head. However, many respondents also noted that inappropriate, or excessive, padding could potentially add compression forces and increase risk of peripheral nerve injury [12]. Intraoperative use of somatosensory evoked potentials (SSEP) has also been shown to have potential benefits in identifying impending nerve injury [13]. SSEP is a technique by which the conduction of a given somatosensory pathway can be monitored during a procedure. Stimuli can be administered, and responses are recorded via electrodes in terms of conduction speed of nerve impulses. Thus, a decrease in the SSEP response is indicative of some impairment of nerve conduction [14]. Changes in upper extremity SSEP conduction in one study were able to be reversed much of the time by changing arm position [13]. This highlights the potential use of SSEP monitoring in the prevention of perioperative nerve injury and further reinforces the importance of proper patient positioning.

It is difficult to quantify the significance of underlying conditions in these cases. None of the patients had a full neurologic examination performed upon their initial presentation due to the emergent nature of their conditions. The owners of case 2 noted upon intake that their dog had been less coordinated and had more difficulty rising in the weeks prior to presentation. While the limited neurologic examination on these patients is understandable, it does limit the ability to assess how much of the subsequent neurologic findings were truly iatrogenic in nature versus preexisting conditions that were exacerbated during the procedure. Both Great Dane patients are believed to have had preexisting conditions that may have affected their gait; one had significant stifle pathology and the other was diagnosed with cervical spondylomyelopathy. It is therefore possible that these patients had some amount of sciatic damage prior to surgery that was subclinical or only mildly evident and was then exacerbated during the procedure, as nerves with chronic dysfunction are more susceptible to perioperative damage [1]. This underscores the fact that, given the emergent nature of the conditions in which these animals were presented, the lack of full neurologic examination limits the ability to conclude how much of the neuropraxia seen postoperatively was a direct result of hospitalization.

The resulting neurologic deficits in the cases described here are likely due to a perfect storm of multiple factors. Potential preexisting conditions, patient characteristics, perioperative positioning, and challenges with postoperative nursing care all contributed to the development of neuropraxia. Possibly the most obvious factor in these cases is that all were giant-breed dogs. This immediately offers some complication in any hospitalization scenario, solely due to the size of these patients and the physical labor required to assist them if they were nonambulatory. The limited ability to help such patients ambulate can result in periods of prolonged recumbency, which may contribute to the development of nerve injury secondary to compression [4].

Recommendations for postoperative nursing care for these patients are obviously labor intensive given their size. Since the presentation of these cases, our hospital has invested in deep bedding and improved staff education in the importance of frequent, regular movement in postoperative management. Deep bedding has been indicated as a potential factor in reducing peripheral nerve injury in downer cows, and the American Society of Anesthesiologists also recommends adequate padding both intraoperatively and in the recovery period to help reduce the risk [4, 12]. The third case was given a crib mattress in the hospital, which may have contributed to the patient's milder clinical manifestations of neuropraxia. Other recommendations include providing adequate support during intraoperative positioning (Figure 1(b)), and consultation with specialists such as those in sports medicine clinicians may be beneficial in generating a set of criteria aimed at identifying patients at high risk of perioperative nerve damage before induction of anesthesia [14]. This set of criteria, in conjunction with an adequate medical history and physical examination, can help to reduce the overall risk to patients.

There is no way to determine the degree to which intraoperative positioning contributed to the development of neurologic deficits in these patients, but these cases do highlight the importance of appropriate management in giant-breed dogs. Based on the knowledge of factors contributing to iatrogenic nerve injury in humans, positioning is but one piece of a complicated puzzle. Preexisting conditions, especially those affecting the nervous system, and patient characteristics also play an important part in developing such undesirable sequelae.

## Data Availability

The clinical data used to support the findings of this study are described within the article.

## References

[1] Warner M. A. (1998). Perioperative neuropathies. *Mayo Clinic Proceedings*.

[2] Dillavou E. D., Roderick Anderson L., Bernert R. A. (1997). Lower extremity iatrogenic nerve injury due to compression during intraabdominal surgery. *The American Journal of Surgery*.

[3] Winfree C. J., Kline D. G. (2005). Intraoperative positioning nerve injuries. *Surgical Neurology*.

[4] Poulton P. J., Vizard A. L., Anderson G. A., Pyman M. F. (2016). High-quality care improves outcome in recumbent dairy cattle. *Australian Veterinary Journal*.

[5] Poulton P. J., Vizard A. L., Anderson G. A., Pyman M. F. (2016). Importance of secondary damage in downer cows. *Australian Veterinary Journal*.

[6] Oosterlinck M., Schauvliege S., Martens A., Pille F. (2013). Postanesthetic neuropathy/myopathy in the nondependent forelimb in 4 horses. *Journal of Equine Veterinary Science*.

[7] Forterre F., Tomek A., Rytz U., Brunnberg L., Jaggy A., Spreng D. (2007). Iatrogenic sciatic nerve injury in eighteen dogs and nine cats (1997–2006). *Veterinary Surgery*.

[8] Bennett D. (1976). An anatomical and histological study of the sciatic nerve, relating to peripheral nerve injuries in the dog and cat. *Journal of Small Animal Practice*.

[9] Flug J. A., Burge A., Melisaratos D., Miller T. T., Carrino J. A. (2018). Post-operative extra-spinal etiologies of sciatic nerve impingement. *Skeletal Radiology*.

[10] Papazoglou L. G., Kazakos G. M., Tsioli V., Zavros N. (2007). What is your diagnosis?. *Journal of Small Animal Practice*.

[11] Saiwai H., Okada S., Kawaguchi K. (2019). Prone position surgery for a professional sumo wrestler with thoracic ossification of the posterior longitudinal ligament resulting in intraoperative brachial plexus injury by hypertrophic pectoral muscles. *Journal of clinical neuroscience*.

[12] American Society of Anesthesiologists Task Force on Prevention of Perioperative Peripheral Neuropathies (2011). Practice advisory for the prevention of perioperative peripheral neuropathies: an updated report by the American Society of Anesthesiologists Task Force on prevention of perioperative peripheral neuropathies. *Anesthesiology*.

[13] Kamel I. R., Drum E. T., Koch S. A. (2006). The use of somatosensory evoked potentials to determine the relationship between patient positioning and impending upper extremity nerve injury during spine surgery: a retrospective analysis. *Anesthesia and Analgesia*.

[14] Bouyer-Ferullo S. (2013). Preventing perioperative peripheral nerve injuries. *AORN Journal*.

